# Supplementary material for: Towards Picogram Detection of Superparamagnetic Iron-Oxide Particles Using a Gradiometric Receive Coil
Source: Sci Rep. 2017 Jul 31;7:6872. doi: 10.1038/s41598-017-06992-5 (PMC5537232; doi:10.1038/s41598-017-06992-5)
Supplement: Supplementary file 4 — Supplementary information [file 41598_2017_6992_MOESM4_ESM.pdf]

Towards Picogram Detection of Superparamagnetic Iron-Oxide Particles Using a Gradiometric Receive Coil

Matthias Graeser[1,*], Tobias Knopp[2,3], Patryk Szwargulski[2,3], Thomas Friedrich[1], Anselm von Gladiss[1], Michael Kaul[4], Kannan M Krishnan[5], Harald Ittrich[4], Gerhard Adam[4], and Thorsten M. Buzug[1]

[1]Institute of Medical Engineering, University of Lübeck, Lübeck, Germany
[2]Section for Biomedical Imaging, University Medical Center Hamburg-Eppendorf, Hamburg, Germany
[3]Institute for Biomedical Imaging, Hamburg University of Technology, Hamburg, Germany
[4]Department for Diagnostic and Interventional Radiology and Nuclear Medicine, University Medical Center Hamburg-Eppendorf, Hamburg, Germany
[5]Department of Materials Science and Department of Physics, University of Washington, Seattle, USA
[*]ma.graeser@uke.de

S Supplementary Information

S.1 Videos

Video S.1. Reconstructed and animated image series of the moving particle sample for the preinstalled receive chain and the gradiometer receive chain (see main article figure 6). Each image is normalized to its current maximum intensity. With decreasing iron content the reconstructed sample appears blurred which is caused by fewer frequencies involved in the reconstruction due to the adapted SNR threshold. For the preinstalled receive chain the detection limit is reached at about 160 ng iron content. For the gradiometer receive chain the detection limit is improved by a factor of 32 to 5 ng iron content.

Video S.2. Reconstructed and animated image series of moving particle samples of different concentration (see main article figure 7). Each sample has the same iron mass of 10 ng but a volume ranging from 1 µL until 128 µL. The resulting concentrations range from 78 µg/L to 10 mg/L (1.4 µmol/L to 178 µmol/L). The detection limit is reached at about 156 µg/L (2.8 µmol/L).

Video S.3. 4D *in vivo* animated reconstruction results of the bolus experiment with the highest concentration (51.2 µg / 10 µL) at a time resolution of 21.41 ms (see main article figure 9). Shown are selected image fusions that reveal how the bolus passes through the mouse heart. The white box inside the images indicate the MPI FOV. Additionally, at the center the temporal progression of the signal in different selected structures (vena cava (top), right atrium and right ventricle (middle), left atrium and left ventricle (bottom)) are shown.

S.2 Equations

In this section, we derive the approximitive sensitivity law outlined in the main article section 2.2. We consider the coil sensitivity of a solenoid at the center (**O**) to be

$$p_x(\mathbf{O}) = \frac{N\mu_0}{l\sqrt{\frac{4R^2}{l^2}+1}}. \tag{1}$$

The inductance of a short solenoid can be calculated to be[1]

$$L = \frac{\mu_0 N^2 \pi R^2}{l+0.9R}. \tag{2}$$

The coil efficiency ρ can then be written as

$$\rho = \frac{p_x(\mathbf{O})}{\sqrt{L}} = \frac{\frac{N\mu_0}{l\sqrt{\frac{4R^2}{l^2}+1}}}{\sqrt{\frac{N^2\mu_0 A}{l+0.9R}}}. \tag{3}$$

With $l = 1.3R$ this can be further simplified to

$$\rho = \frac{\frac{N\mu_0}{2.385R}}{N\sqrt{\frac{\mu_0 \pi R^2}{2.2R}}} = \frac{\sqrt{2.2\cdot\mu_0}}{2.385R\sqrt{R\pi}} \propto \frac{1}{\sqrt{R^3}}. \tag{4}$$

Thus, the sensitivity scales inversely with $R^{1.5}$.

References

1. Wheeler, H. A. Simple inductance formulars for radio coils. *Proc. IRE* **16**, 1398 (1928).
